# Vaccine Virus

**Published:** 1874-12

**Authors:** 


*Vaccine Virus.*

We take pleasure in recommending Dr. E. Cutter & Brother, Lexington, Mass., as reliable parties from whom to obtain pure vaccine virus. We have tried samples from them and find it genuine. See their advertisement, and remind your druggist to order from them.

